# The Novel Antigenic Epitopes of African Swine Fever Virus Inner Membrane p54 Protein Revealed by Monoclonal Antibodies

**DOI:** 10.3390/ani15091296

**Published:** 2025-04-30

**Authors:** Jiajia Zhang, Kaili Zhang, Shaohua Sun, Ping He, Dafu Deng, Hanrong Lv, Mingwang Xie, Pingping Zhang, Wanglong Zheng, Nanhua Chen, Jianfa Bai, Jianzhong Zhu

**Affiliations:** 1 College of Veterinary Medicine, Yangzhou University, Yangzhou 225009, China; 2 Joint International Research Laboratory of Agriculture and Agri-Product Safety, Yangzhou University, Yangzhou 225009, China; 3 Comparative Medicine Research Institute, Yangzhou University, Yangzhou 225009, China; 4 Jiangsu Co-Innovation Center for Prevention and Control of Important Animal Infectious Diseases and Zoonoses, Yangzhou University, Yangzhou 225009, China; 5 Kansas State Veterinary Diagnostic Laboratory, Kansas State University, Manhattan, KS 66506, USA

**Keywords:** African swine fever virus, structural protein p54, monoclonal antibodies, antigenic epitope, ELISA detection

## Abstract

African swine fever (ASF) is caused by the African swine fever virus (ASFV); infection in domestic pigs and wild boars leads to a highly contagious, hemorrhagic disease. The p54 protein is encoded by the ASFV E183L gene and is an important structural protein located on the inner envelope of the virus. It is involved in processes of virus assembly, apoptosis induction, and neutralizing antibody production. In this study, three specific monoclonal antibodies (mAbs) against ASFV p54 protein were generated, namely 6B11, 3E3, and 3C10, from mice who were immunized with recombinant prokaryotic p54-truncated protein. Three novel linear B cell epitopes, recognized by the mAbs, were revealed: ^60^AAIEEEDIQFINP^72^, ^128^MATGGPAAAPAAASAPAHPAE^148^, and ^163^MSAIENLRQRNTY^175^. The epitopes ^60^AAIEEEDIQFINP^72^ and ^163^MSAIENLRQRNTY^175^ were highly conserved in genotype I and II ASFV strains. In addition, the epitope peptide ELISA can be used for the detection of ASFV antibodies. Our work provides new insights for p54 antigenicity and an alternative tool for serological diagnosis of ASF.

## Simple Summary

African swine fever is a highly contagious disease that affects domestic pigs and wild boars. Despite the serious effects on the pig industry worldwide, there are no currently available effective vaccines. Therefore, understanding this pathogen is necessary for the control and prevention of African swine fever. The pathogenic virus expresses more than 150 viral proteins, with the p54 protein located on the inner envelope and an important structural protein. Here, we developed three monoclonal antibodies specific for the p54 protein, and identified three precise antigenic epitopes recognized by these monoclonal antibodies. A comparison with the p54 sequences of different viral strains revealed that some of these antigenic epitopes are highly conserved in genotype I and II strains. Moreover, the anti-genic epitopes can be used for immune detection of specific viral antibodies. Thus, our work provides new insights into the virus’s antigenicity and offers an alternative tool for the diagnosis of African swine fever infection.

## 1. Introduction

African swine fever (ASF) is a highly contagious disease caused by African swine fever virus (ASFV), with the main susceptible animals being domestic pigs and wild boars [1]. The symptoms of diseased pigs include elevated body temperature, respiratory disorders, neurological symptoms, and other clinical symptoms [2]. The mortality rate of pigs who are infected with virulent strains is as high as 100% [3]. ASFV was first reported in Kenya in 1921 and was introduced to Georgia in 2007 [4,5]. In August 2018, ASF first appeared in Shenyang, Liaoning Province, China, and quickly spread to various parts of the country [6]. Currently, ASFV has spread into different countries in Asia, Europe, and Africa [7]; however, there is still a lack of effective commercial vaccines and antiviral drugs. Therefore, establishing efficient ASF detection is of great significance for the prevention and control of ASF.

ASFV is a double-stranded DNA virus with a symmetrical icosahedral structure and a diameter of approximately 260–300 nm [8]. The ASFV genome has a total length of 170 kb to 190 kb, encoding 150–200 viral proteins, which can be divided into over 60 structural proteins and over 100 non-structural proteins [9]. The p54 protein is an important structural protein encoded by the E183L gene, with a relative molecular weight of 24–28 kD. It is located in the inner envelope of viral particles and appears in the late stage of viral infection as a late-stage protein [10]. The term “p54” is not related to its molecular weight (about 25 kD), but to its relative position in the two-dimensional gel [11]. The viral p54 protein can rely on its transmembrane structure to transform the endoplasmic reticulum (ER) membrane into a viral inner membrane precursor and thus play an important role in the process of viral particle assembly [10]. There is a segment of dynein-binding domains (DBDs) within amino acids 149-161 in the C-terminus of p54 protein [12]. After the virus enters the cell, p54 interacts with the intracellular dynein to promote the movement of virus particles in the cytoplasm [12,13]. In addition, the DBD is involved in activating caspase-3, leading to apoptosis [14].

ASFV-infected pigs produce strong antibody responses to the highly conserved ASFV structural proteins p54, p30, and p72, thus suggesting that these proteins are the ideal antigens for developing serological assays [9,15]. After ASFV infection, the anti-p54 antibodies appeared as early as 8 days post infection, and the high levels of antibodies persisted for several weeks [16]. During the screening and identification of dominant antigens of ASFV, p54 and p30 were presented as the major antigens reacting with ASFV-positive sera [17]. ELISA antibody detection based on p54 protein has the same performance as the WOAH standard ELISA assay based on whole-virus antigens [18]. Due to its strong immunogenicity, the p54 protein has often been used as a target in the development of serological diagnoses [17,19,20,21,22,23].

In this study, we expressed and purified p54-truncated protein, immunized mice, and generated three monoclonal antibodies (mAbs). The novel antigenic epitopes recognized by the three mAbs were identified, namely ^60^AAIEEEDIQFINP^72^, ^128^MATGGPAAAPAAASAPAHPAE^148^, and ^163^MSAIENLRQRNTY^175^. In addition, an epitope-based indirect ELISA was preliminarily developed, which can specifically detect ASFV-positive serum.

## 2. Materials and Methods

### 2.1. Mice, Cells, Sera, and Viruses

BALB/c mice of 6 to 8 weeks were purchased from the experimental animal facility of Yangzhou University. HEK-293T cells (ATCC# CRL-3216), Marc-145 cells (Ubigene# YC-A070), and myeloma SP2/0 cells (ATCC# CRL-1581) were cultured in Dulbecco modified Eagle medium (DMEM, Hyclone Laboratories, Logan, UT, USA) containing 10% fetal bovine serum (FBS, Eallbio, Beijing, China). Primary porcine alveolar macrophages (PAMs) were cultured in RPMI 1640 medium (Hyclone Laboratories) containing 2% FBS. The ASFV strain YZ-1 (genotype II, GenBank accession No. 456300) was kept in the facility of Yangzhou University Animal Biosafety Level 3 (ABSL-3), certified by the Ministry of agriculture and rural affairs (0714002001109-1) [24]. The porcine reproductive and respiratory syndrome virus (PRRSV), recombinant PRRSV expressing p54 (PRRSV-p54), porcine epidemic diarrhea virus (PEDV), swine influenza virus (SIV), and various porcine serum samples were stored in our lab. Animal experiments were conducted in strict accordance with the Guide for the Care and Use of Laboratory Animals of Yangzhou University (SYXK(JS)-2022-0044).

### 2.2. Expression and Purification of p54 Truncation Protein

The truncated ASFV p54 gene (p54 JD, 55-184 aa) was obtained by PCR amplification using the primers shown in Table S1 from the pENTR4-ASFV p54-2HA plasmid constructed and stored in our laboratory. The PCR product was cloned into the *Sal*I and *Xho*I sites of the pET28a-6His vector through Seamless/In-Fusion Cloning, and the sequence was confirmed by Sanger DNA sequencing. The recombinant pET28a-p54 JD-6His was transformed into BL21-competent *E. coli* cells to explore the induction conditions by 1 mM IPTG, including the time and temperature. Under optimal induction, the bacteria were collected by centrifugation, resuspended in PBS, and sonicated on ice. After centrifugation, the bacterial pellet was resuspended in PBS solution containing 8 M urea, shaken overnight at 4 °C, and then subjected to gradient dialysis and subsequent refolding.

### 2.3. Generation of Anti-p54 Monoclonal Antibodies (mAbs)

Mice were immunized with purified p54 protein plus adjuvant Montanide gel (SEPPIC SA, Cedex, France), following the same procedure as we described before [25,26]. Five days after the three immunizations, blood samples were collected from the tail vein of the mice, and the serum titer was determined by ELISA and confirmed by Western blotting. The mice with the highest titer of serum ELISA antibody were used for the final booster, and splenocytes from the mice were harvested and fused with SP2/0 cells following the standard procedure. Hybridomas secreting p54-specific antibodies were screened out by p54-coated indirect ELISA and confirmed by Western blotting. Positive hybridomas were subcloned three times by limiting the dilution and confirmed by antibody production. Mouse ascites were prepared using sterile liquid paraffin and subcloned hybridoma following the standard procedure.

### 2.4. Mapping of the Antigenic Epitopes of the p54 Protein

According to the epitope prediction results, the full-length p54 protein was first divided into three fragments (1-54aa, 55-122aa, 123-184aa), cloned into the eukaryotic expression vector pEGFP-N1, and transfected into 293T cells. The truncated protein fragments recognized by p54 mAbs were identified by Western blotting. According to the results, the identified p54 protein fragments were further divided and examined for recognition by p54 mAbs. Subsequently, the p54 protein fragments were progressively shortened from both ends to clarify the minimal antigenic epitopes targeted by p54 mAbs using Western blotting. A total of 28 p54 fragments (P1–P28) were PCR-amplified and cloned into pEGFP-N1 using Seamless Cloning/In-Fusion Cloning, with all the cloning primers being listed in Table S1.

### 2.5. Western Blotting (WB) and Immunofluorescence Assay (IFA)

The protein samples were mixed with 4 × loading buffer at a ratio of 3:1, heated at 100 °C for 5–10 min, and run by 8–10% SDS-PAGE. The gel was stained with Coomassie blue staining or detected by immunoblotting as we described previously [25,27], with anti-His mouse mAb (Transgen Biotech, Beijing, China), ASFV-positive pig serum, and p54 mAbs as the primary antibodies and HRP-conjugated Goat anti-mouse IgG (1:10,000, BBI, Shanghai, China) or HRP-conjugated Goat anti-pig IgG (1:10,000, Proteintech, Wuhan, China) as the secondary antibody.

The 293T cells in 12-well cell culture plates (2 × 10^5^ cells/well) were transfected with pCAGGS-p54-2HA and control pCAGGS-2HA for 24 h. Marc-145 cells in 12-well cell culture plates (2 × 10^5^ cells/well) were mock infected or infected with recombinant PRRSV-p54 virus (MOI = 1) for 72 h. The cells were fixed, permeabilized, and stained with ascites p54 mAbs and Goat anti-mouse IgG H&L Alexa fluor 594 (1:500, Abcam, Shanghai, China). After DAPI staining (1:500, Abcam, Shanghai, China), the stained samples were visualized using a fluorescence microscope (Olympus Corporation, Tokyo, Japan).

### 2.6. ASFV p54 Protein Indirect ELISA and Antigenic Peptide Indirect ELISA

For the p54 protein indirect ELISA, the purified p54 JD fusion protein was diluted in PBS (0.625 μg/mL) as the coating antigen. For the epitope-mediated indirect ELISA, the synthesized epitope peptide was diluted in PBS (0.3125–10 μg/mL) as the coating antigen. Both indirect ELISAs followed the same procedures as we described previously [25,27]. After termination by 2 M H_2_SO_4_, the OD_450nm_ value of each well was determined. The ratios of hybridoma supernatant versus negative supernatant and positive sera versus negative serum (P/N) were calculated, with P/N ≥ 2.1 as positive.

### 2.7. Bioinformatics Analysis

The hydrophobic region and transmembrane region of the p54 protein were analyzed using the TMHMM-2.0 tools (https://services.healthtech.dtu.dk/services/TMHMM-2.0/) and an ExPASY Prot scale (https://web.expasy.org/protscale/), respectively. The prediction of antigenicity was predicted via an online website (http://tools.iedb.org/main/bcell/). The p54 antigenic epitopes were aligned with the p54 sequences of 170 ASFV strains from GenBank by Clustal W in Megalign version 7.1.0 (DNAStar). The spatial structure of p54 protein was predicted by Alphafold2 (https://colab.research.google.com/github/sokrypton/colabFold/blob/main/Alphafold2.ipynb). The analysis of distribution and structure of the epitopes in the p54 protein were performed by the PyMOL molecular graphics system (version 2.4.0, Schrödinger, LLC, New York, NY, USA).

## 3. Results

### 3.1. Production and Identification of Recombinant p54 Truncation Protein

**Figure 1:**
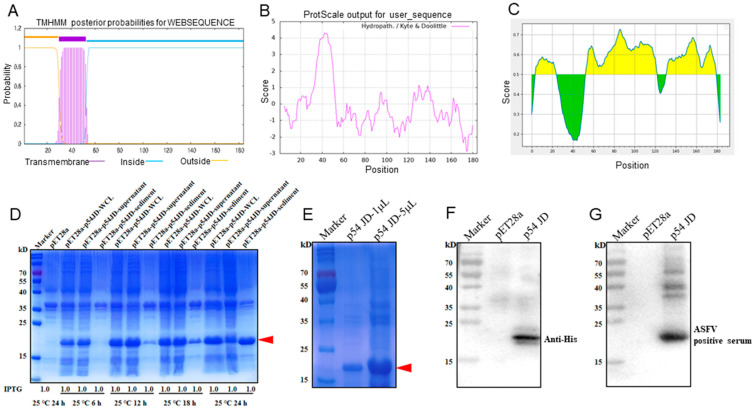
Production and identification of the p54-truncated fusion proteins. (**A**–**C**) Prediction of the transmembrane (**A**), hydrophilicity (**B**), and antigenic regions (**C**) of the p54 protein using the online tools described in the Materials and Methods section. In panel (**C**), the yellow and green represent the regions of high and low antigenicity, respectively. (**D**) The p54 JD was induced by 1 mM IPTG for 6 h, 12 h, 18 h, and 24 h at 25 °C, with the empty pET28a vector-transformed bacteria being used as a control. Whole bacterial lysates, supernatants, and sediments were examined for p54 JD protein expressions by SDS-PAGE and Coomassie blue staining. (**E**) The purified p54 JD protein was confirmed by SDS-PAGE and Coomassie brilliant blue staining. The p54 JD of 20 kD is indicated by arrow heads. (**F**,**G**) The purified p54 JD protein was verified by Western blotting using anti-His mAb (**F**) and ASFV-positive pig serum (**G**), respectively. The lysates of bacteria transformed with the empty pET28a vector were used as controls.

ASFV p54 protein is a transmembrane protein [9,10]. As expected, the bioinformatics analysis showed that the ASFV p54 protein possesses a highly hydrophobic transmembrane domain (TM) of 23 amino acids (30-52aa) near the N-terminal end (Figure 1A,B). Because a protein with a transmembrane domain is difficult in terms of expression and the C-terminal intramembrane region contains more antigenicity (Figure 1C), the C-terminal intramembrane domain (p54 JD, 55-184aa) was chosen for expression. The p54 JD-encoding sequence was cloned into a pET28a vector, and the recombinant protein was expressed in *E.coli* BL21 cells. The SDS-PAGE results showed that the p54 JD protein was obviously induced by 1 mM IPTG at 25 °C or 37 °C for 24 h, appearing as 20 kD bands (Figure S1A). At 25 °C, under the induction of 1 mM IPTG for 24 h, the p54 JD protein was expressed in both a soluble form and in an inclusion body, and the bands were relatively separate at 25 °C (Figure 1D). The purified p54 JD from the inclusion body presented as one major protein band in the SDS-PAGE, with a molecular weight about 20 kD (Figure 1E). The purified p54 JD protein could be recognized by anti-His mAb (Figure 1F) and by ASFV-positive pig serum (Figure 1G). After three immunizations, the serum antibody titer in mice reached above 1:409,600, indicating that the p54 recombinant protein has good immunogenicity (Figure S1B).

### 3.2. Generation of Specific Monoclonal Antibodies (mAbs) for p54 Protein

**Figure 2:**
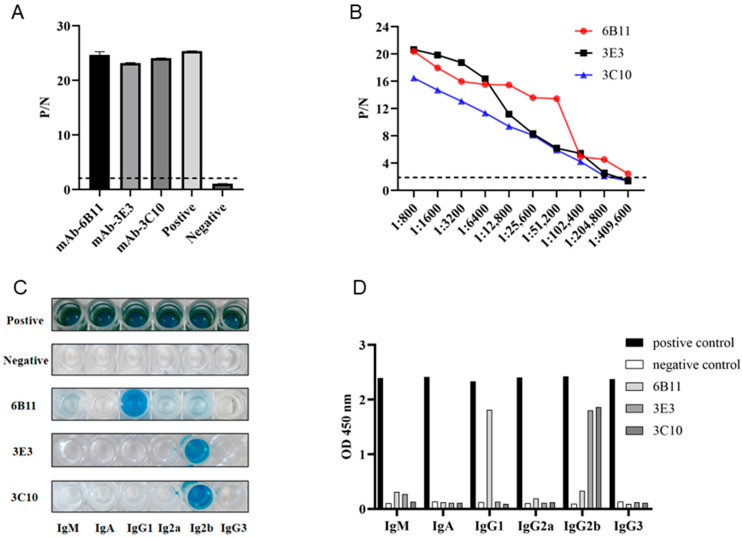
Preparation and characterization of anti-p54 monoclonal antibodies (mAbs). (**A**) The reactivity of mAbs was tested in p54 JD protein-coated indirect ELISA. The cell supernatants of hybridoma clones were used as the primary antibodies, the SP2/0 cell supernatant was used as the negative control, and the serum of immunized mice was used as a positive control. (**B**) Measurement of antibody titers of the ascite MAbs 6B11, 3E3, and 3C10 by the p54 JD ELISA. The dotted lines denote the P/N values of 2.1. (**C**,**D**) Identification of the mAb subtypes using the monoclonal antibody subclass identification kit (C060101) purchased from CELLWAY-LAB (Louyang, China), following the product manual. The color development image (**C**) and OD_450nm_ quantification graph (**D**) are presented.

Monoclonal antibodies (mAbs) were obtained by a standard cell fusion protocol. After 6–8 days of fusion, the cell supernatants of the hybridomas were collected and used for screening of p54 mAbs by indirect ELISA. Through screening and three subsequent subcloning processes, three p54 mAb clones, named 6B11, 3E3, and 3C10, were obtained (Figure 2A). Indirect ELISA was utilized to measure the affinity of the three mAbs to purified p54 protein. As shown in Figure 2B, the highest affinity of clone 6B11 was 1:409,600, and those of clones 3E3 and 3C10 were 1:204,800. The antibody subclass identification showed that the mAb 6B11 was IgG1, and the mAbs 3E3 and 3C10 were both IgG2b (Figure 2C,D).

**Figure 3:**
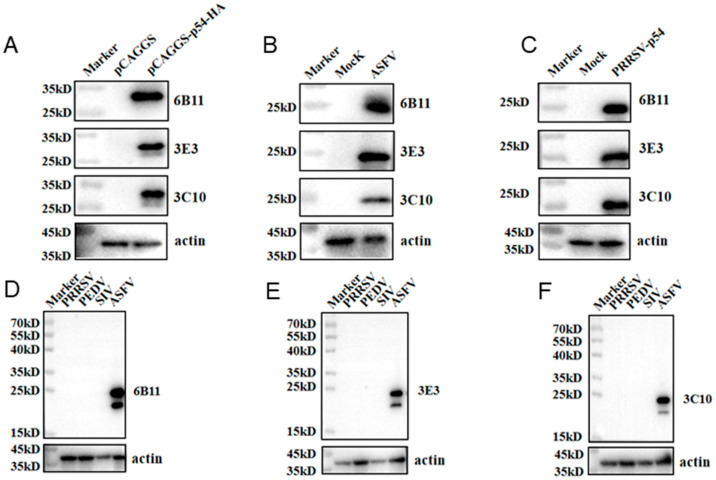
Specific reactivity of the p54 mAbs, determined using Western blotting. (**A**) The 293T cells were transfected with pCAGGS-p54-2HA and a pCAGGS-2HA vector control, respectively, for 24 h. (**B**) The primary PAMs were mock-infected or infected with ASFV (MOI 0.1) for 96 h. (**C**) Marc-145 cells were mock-infected or infected with PRRSV-p54 (MOI 0.1) for 96 h. The cells were collected, and cell lysates were detected for p54 expressions by Western blotting with the mAbs 6B11, 3E3, and 3C10 as primary antibodies. (**D**–**F**) Western blotting of PRRSV-, PEDV-, and SIV-positive samples with three p54 mAbs, 6B11 (**D**), 3E3 (**E**), and 3C10 (**F**), with an ASFV-positive sample as the positive control.

**Figure 4:**
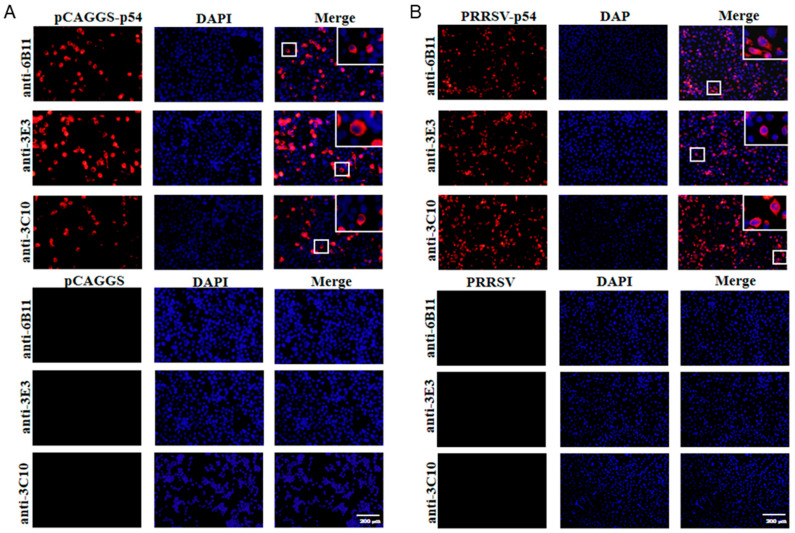
The specific reactivity of the p54 mAbs, analyzed with immunofluorescence assay. (**A**) The 293T cells were transfected with pCAGGS-p54-2HA and pCAGGS-2HA vectors, respectively, for 24 h. (**B**) Marc-145 cells were infected with PRRSV-p54 (MOI 0.1) or mock-infected as controls for 72 h. Cells were fixed and stained with 6B11, 3E3, or 3C10 mAb, together with Goat anti-mouse IgG H&L Alexa Fluor 594. Cellular nuclei were counterstained with 4′,6′-diamidino-2-phenylindole (DAPI). The small box areas are magnified 10× and placed at the upper-right corners. The negative controls of pCAGGS-2HA vector-transfected 293T cells in panel A and mock-infected Marc-145 cells in panel B are presented at the low parts. Scale bars, 200 μm.

The prepared ascite p54 mAbs were used as the primary antibodies to detect the specific reactions with different types of p54 proteins in Western blotting. The p54 proteins included those in 293T cells that were transfected with pCAGGS-p54-2HA, in primary PAMs that were infected with ASFV, and in Marc-145 that was infected with recombinant PRRSV-p54. The results showed that the three mAbs 6B11, 3E3, and 3C10 all reacted specifically with different types of p54 proteins (Figure 3A–C). Further, the three p54 mAbs were tested in Western blotting for their reactivity with different porcine viruses, including porcine reproductive and respiratory syndrome virus (PRRSV), porcine epidemic diarrhea virus (PEDV), and swine influenza virus (SIV). The results showed that the three p54 mAbs only specifically recognized ASFV, but not PRRSV, PEDV, and SIV (Figure 3D–F). Additionally, the immunofluorescence assay showed that the three p54 mAbs had specific reactions with eukaryotic p54 in plasmid-transfected cells (Figure 4A) and in PRRSV-p54-infected cells (Figure 4B), with the expressed p54 mainly being localized in the cytoplasm.

### 3.3. Identification of the Precise Antigenic Epitopes Targeted by p54 mAbs

**Figure 5:**
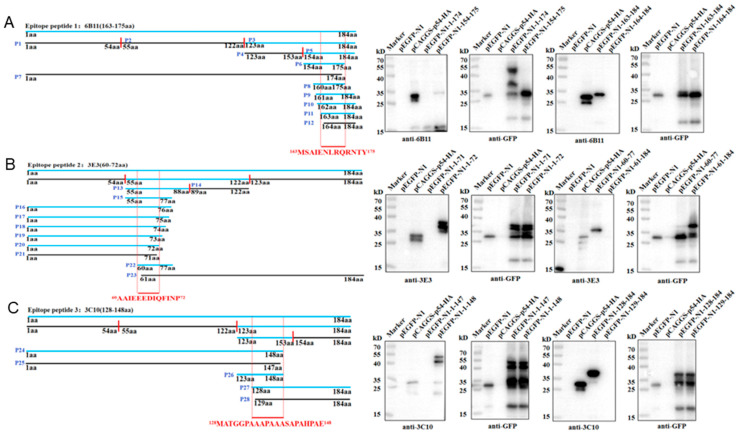
Identification of the antigenic epitopes that are recognized by p54 mAbs. The left parts are the schematic diagrams of p54 and its truncated fragments. The blue p54 and fragments denote reactivity with mAbs, whereas the black p54 fragments denote non-reactivity with mAbs. The smallest epitopes that are recognized by mAbs 6B11 (**A**), 3E3 (**B**), and 3C10 (**C**) are colored in red and position-labeled. The aa is an abbreviation for amino acid. The right parts are the Western blotting analysis of the critical C-terminal amino acid (**left**) and N-terminal amino acid (**right**) for p54 reactivity with mAbs 6B11 (**A**), 3E3 (**B**), and 3C10 (**C**), respectively.

The antigenic epitopes that were recognized by the three p54 mAbs were determined by Western blotting. According to the prediction results of antigenic epitopes in Figure 1C, the full-length p54 protein was separated into three fragments (P1, 1-54aa; P2, 55-122aa; P3, 123-184aa), and the recombinant plasmids were constructed and transfected in 293T cells for truncation protein expressions. The results showed that 6B11 (Figure 5A) and 3C10 (Figure 5C) recognize the P3 region, and 3E3 (Figure 5B) recognizes the P2 region. The P2 and P3 regions were further separated into two fragments (P13 and P14; P4 and P5), and it turned out that 6B11, 3E3, and 3C10 recognize P5, P13, and P4, respectively. Based on information on the reactive fragments, the p54 protein was gradually shortened from both ends until it could not react with the p54 mAbs, in order to determine the smallest epitopes that are recognized by p54 mAbs. After rounds of shortening and testing, the final results showed that 6B11 recognizes the epitope ^163^MSAIENLRQRNTY^175^ (Figure 5A), 3E3 recognizes the epitope ^60^AAIEEEDIQFINP^72^ (Figure 5B), and 3C10 recognizes the epitope ^128^MATGGPAAAPAAASAPAHPAE^148^ (Figure 5C).

**Figure 6:**
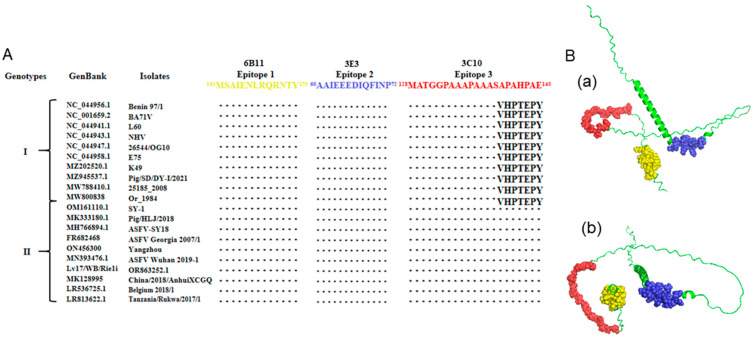
Sequence conservation analysis of the identified epitopes across different genotypes of ASFV strains. (**A**) Alignment of the p54 protein sequences of representative genotypes I and II of ASFV strains with the three antigenic epitopes that were identified (epitope 1, ^163^MSAIENLRQRNTY^175^; epitope 2, ^60^AAIEEEDIQFINP^72^; and epitope 3, ^128^MATGGPAAAPAAASAPAHPAE^148^). The GenBank accession number and isolate name of each ASFV strains are indicated. The dotted area indicates the identical conserved amino acid sequences; otherwise, the amino acids are marked. (**B**) Localization of the three recognized mAb epitopes in the structure of ASFV p54 protein (green). Antigenic epitope 1 is in yellow, epitope 2 is in blue, and epitope 3 is in red in the front view (**a**) and bottom view (**b**).

To determine the conservation of the three identified p54 antigenic epitopes, 170 ASFV E183L gene sequences were downloaded from GenBank, and the encoded protein sequences were aligned with the three antigenic epitope sequences. The results showed that the two antigenic epitopes of mAbs 6B11 and 3E3 were highly conserved across genotype I and genotype II ASFV strains, and the one of 3C10 was highly conserved in genotype II ASFV strains (Figure 6A and Table S2). The p54 protein structure predicted by Alphafold2 is composed of a α helix and random coils. Among the three epitopes, one is located in the α helix, and two are inside the random coils (Figure 6B).

### 3.4. Validation of the p54 Epitopes by Competitive ELISA and Establishment of Indirect Epitope ELISA for Detection of ASF Antibody

**Figure 7:**
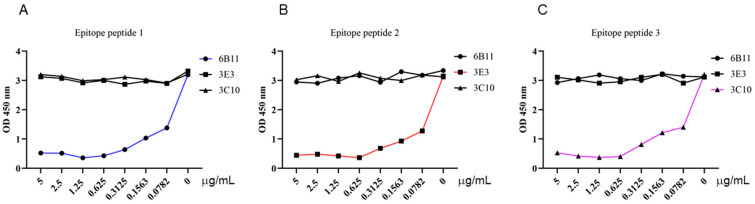
Competitive ELISA validation of the epitope peptides that were recognized by the p54 mAbs. In the p54 JD protein-coated indirect ELISA, three synthesized short peptides were diluted at the indicated concentrations (μg/mL) and combined with the primary antibodies of three ascite mAbs, 6B11 (**A**), 3E3 (**B**), and 3C10 (**C**), for competitive binding with mAbs. The groups without short peptides were set as the negative controls. Finally, based on the OD_450_ results in the ELISA, the graphs were drawn, and the correct epitopes were determined.

To further determine whether the three identified epitopes are B cell epitopes that are recognized by monoclonal antibodies, we synthesized three epitope peptides and applied them in the p54 JD-coated indirect ELISA for competitive binding. The competitive ELISA results demonstrated that the synthesized 6B11, 3E3, and 3C10 epitope peptides can inhibit the reaction of the corresponding mAbs with a coated antigen of the p54 JD protein, in dose-dependent manners, but the cannot inhibit the reaction of the other two p54 mAbs with the p54 JD protein. These results further verified the correctness of the epitope peptides that were recognized by the p54 mAbs (Figure 7A–C).

**Figure 8:**
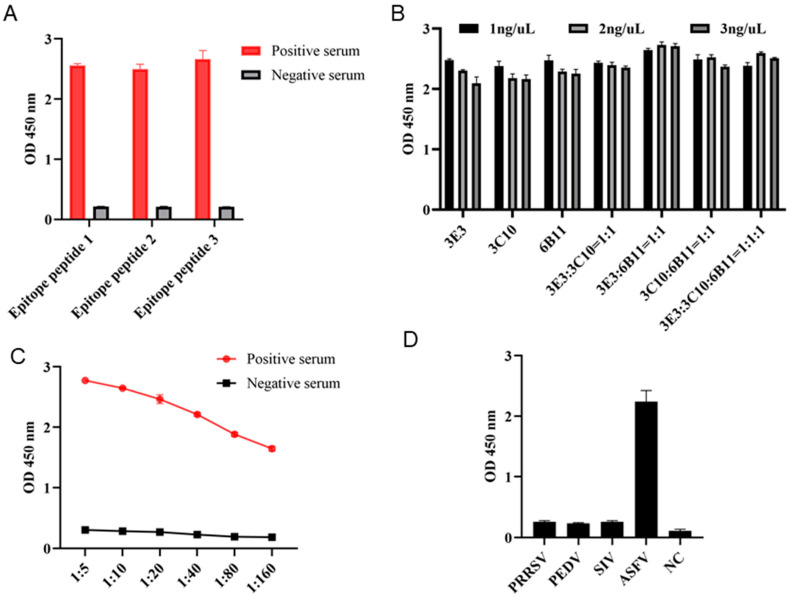
Development of indirect epitope ELISA for detecting ASF-specific antibodies. (**A**) The p54 epitope peptides were used for coating at a concentration of 1 ng/μL. The epitope-based ELISAs were each tested for detection of ASFV-positive and -negative sera, with a serum dilution of 1:10. (**B**) The coating epitope combinations and concentrations in indirect ELISA for the detection of ASFV-positive serum (1:10 dilution). (**C**) The serum dilutions in the indirect ELISA with coating of a peptide 1 and 2 combination and a concentration of 2 ng/μL. (**D**) The specificity of the established indirect epitope ELISA. PRRSV-, PEDV-, SIV-, and ASFV-positive pig sera and a negative pig serum were used.

In order to determine whether the three epitopes can be used for diagnosis, the three epitope peptides were each used as the coated antigen with a concentration of 1 ng/μL, and the ASF-positive and -negative sera were diluted at 1:10 for indirect ELISA detection of the serum antibody. The results showed that all the three epitope peptides can be used for detection of antibodies in ASF-positive serum (Figure 8A). Subsequently, the combination of epitope peptides in the indirect ELISA was explored by the checkerboard method. The results showed that the highest OD_450_ values can be obtained with the coating peptide combination of 6B11:3E3 = 1:1 and a total concentration of 2 ng/μL (Figure 8B). With the optimal coating peptides, we continued to explore the optimal dilution of serum. The 1:5 dilution of ASF-positive serum was shown to give the best performance (Figure 8C). The established epitope ELISA was used to detect antibodies in ASFV-, PRRSV-, PEDV- and SIV-positive pig sera, and only the ASFV-positive serum was successfully detected, indicating high specificity (Figure 8D).

## 4. Discussion

Since the outbreak of ASF in Kenya in the 1920s, the virus has had a serious impact on the global pig industry [28]. Despite the extensive research that has been carried out on ASF, there is still a lack of effective vaccines or antiviral strategies, and the control of ASF mainly relies on strict hygiene measures [28]. Further, the emergence of low-virulence natural mutants has brought greater difficulties to the early diagnosis of ASF and new challenges to ASFV control [29]. Therefore, the in-depth study of the mechanism of pathogenesis and immune evasion of ASFV and establishment of detection methods for early detection and rapid diagnosis are of great significance for the prevention and control of ASF.

Since the outbreak of ASF, researchers have prepared a number of mAbs against different proteins of ASFV and used the mAbs to establish a variety of ASF detection methods, providing a strong technical support for the detection of ASF [27,30,31,32]. The ASFV structural protein p54, encoded by E183L, is a good target for ASF detection and vaccine development [15,33,34]; however, the expression efficiency of the full-length p54 is poor due to the feature of a transmembrane protein. Therefore, we discarded the hydrophobic transmembrane region and chose the intramembrane region with high antigenicity for expression. This greatly improved the expression efficiency in the BL21 prokaryotic system, and a large amount of immunogenic p54 protein was obtained. The three generated p54 mAbs could be successfully used for various immunological experiments, including ELISA, WB, and IFA, demonstrating a broad spectrum of applications.

B cell antigenic epitopes are key factors that determine the antigenicity of viral structural proteins and induce humoral immune responses, which is helpful for improving the detection efficiency of detection reagents and the development of subunit vaccines [35], especially for key ASFV antigen proteins such as p30, p72, and p54. In addition to two linear epitopes, 46-60 aa, and one between 149 and 161aa in the dynein-binding region (DBD) [36], previous studies have demonstrated that the p54 protein has different linear epitopes, including 5-9 aa, 10-13 aa, 37-44 aa, 63-72 aa, 65-75 aa, 76-81 aa, 93-113 aa, 103-111 aa, 118-127 aa, 110-118 aa, 112-122 aa, 143-152 aa, and 175-184 aa [22,30,37,38,39,40,41,42]. In this study, we identified the three precise p54 epitopes of 60-72 aa, 128-148 aa, and 163-175 aa, which are new antigenic epitopes and different from the above-reported epitopes. These new epitopes all have reactivity with ASFV-positive serum, indicating them as natural antigenic epitopes. The p54 new antigenic epitopes can become a new target to improve the detection of ASF and have a potential application value in the monitoring and control of ASF epidemics.

ASF is mainly distributed in major economic regions with frequent trade in the pig industry [28]. As ASF continues to prevail in a region, ASFV will mutate, causing chronic infection or asymptomatic infection [43,44]. In this case, an accurate serological detection method is needed to detect the antibodies of animals to screen out recessive or asymptomatic infected pigs. Therefore, sensitive and reliable serological diagnostic assays of ASF infection is needed. Theoretically, epitope-based serological detection has an advantage over protein and antigen-based detection, with lower crossreaction and higher specificity [45,46]. Currently, all commercial ELISA kits, including the WOAH-approved ELISA kit detecting ASFV antibodies, are all based on ASFV proteins or live virus as coating antigens [18]. Here, we demonstrated that the combination of 6B11 and 3E3 peptides, mixed at 1:1, as coating antigens can achieve the effective detection of serum ASF antibodies. The antigenic variability of p54 is lower than that of p72; therefore, p54 typing is used as a complementary index for p72 typing of ASFV strains [47]. Despite the antigenicity variation of p54, considering that both antigenic peptides of 6B11 and 3E3 are highly conserved across all genotypes I and II of ASFV strains, the peptide ELISA is useful for the detection of genotype I and II ASFV infections in China. In our study, there was no comparison of epitope ELISA with commercial or WOAH-approved assays, and it lacked the confirmation with ASFV-positive sera from diverse genotypes or geographical regions. Therefore, the developed epitope ELISA assay needs to be verified with these requirements before clinical application.

In summary, three specific mAbs for ASFV p54 protein were generated, and three corresponding new linear B cell epitopes were identified. The identified epitopes ^60^AAIEEEDIQFINP^72^ and ^163^MSAIENLRQRNTY^175^ are highly conserved in genotype I and II ASFV strains. Based on these two peptides, an indirect ELISA detecting p54 antibodies was preliminarily developed, which can potentially be applied potentially for the early detection and rapid diagnosis of ASF, providing an alternative for current commercial ELISA kits detecting ASFV antibodies.

## 5. Conclusions

Three ASFV p54 specific monoclonal antibodies were generated, allowing use in various immunoassays. Three precise antigenic epitopes were identified using the three p54 monoclonal antibodies. The antigenic epitopes can be used for the detection of ASFV antibodies in the ELISA assay.

## Data Availability

The authors confirm that the data supporting the findings of this study are available within the article and its Supplementary Materials.

## Supplementary Materials

The following supporting information can be downloaded at https://www.mdpi.com/article/10.3390/ani15091296/s1: Figure S1. Production of the p54-truncated fusion protein and immunization of mice with purified p54 JD protein. (A) The p54 JD were induced with or without 1 mM IPTG at 16 °C, 25 °C, and 37 °C, respectively. The bacterial lysates, lysate supernatants, and sediments were analyzed by SDS-PAGE and Coomassie blue staining for p54 protein expressions. The p54 JD of 20 kD are indicated by the arrow head. (B) Serum antibody titration of immunized mice was measured in an indirect ELISA coated with different concentrations of the p54 JD protein. Table S1. The cloning PCR primers used in this study. Table S2. The identity of p54 epitope sequences across different genotypes of ASFV strains. File S1. WB raw data.
