# Supplementary material for: The Novel Antigenic Epitopes of African Swine Fever Virus Inner Membrane p54 Protein Revealed by Monoclonal Antibodies
Source: Animals (Basel). 2025 Apr 30;15(9):1296. doi: 10.3390/ani15091296 (PMC12070866; doi:10.3390/ani15091296)
Supplement: Supplementary file 1 [file animals-15-01296-s001.zip › Figure S1.pdf]

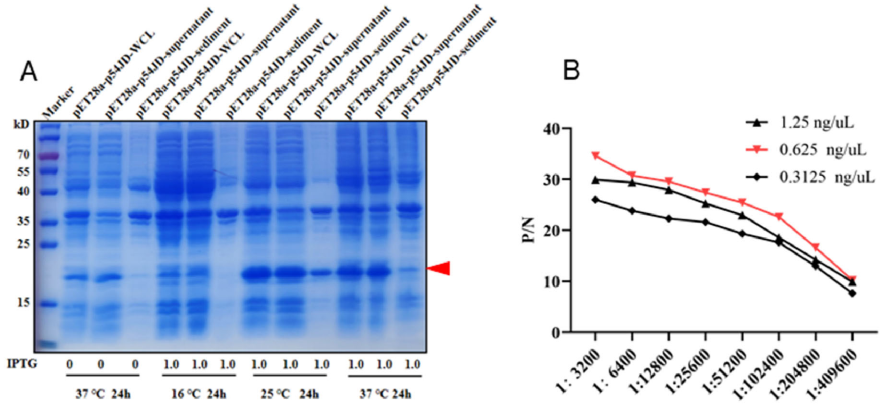

Fig S1

Figure S1. Production of the p54-truncated fusion protein and immunization of mice with purified p54 JD protein. (**A**) The p54 JD were induced with or without 1 mM IPTG at 16 °C, 25 °C, and 37 °C, respectively. The bacterial lysates, lysate supernatants, and sediments were analyzed by SDS-PAGE and Coomassie blue staining for p54 protein expressions. The p54 JD of 20 kD are indicated by the arrow head. (**B**) Serum antibody titration of immunized mice was measured in an indirect ELI-SA coated with different concentrations of p54 JD protein.
