# Supplementary material for: The Novel Antigenic Epitopes of African Swine Fever Virus Inner Membrane p54 Protein Revealed by Monoclonal Antibodies
Source: Animals (Basel). 2025 Apr 30;15(9):1296. doi: 10.3390/ani15091296 (PMC12070866; doi:10.3390/ani15091296)
Supplement: Supplementary file 1 [file animals-15-01296-s001.zip › Table S1.pdf]

Supplementary Table S1. The cloning PCR primers used in this study.

p54 fragments	Sequences (5’ - 3’)
pCAGGS-p54-2HA	F:tctcatcattttggcaaa*GAATTC***atg**gatagcgagttttttc
	R:catcgtatgggtagctggt*GATATC*taaagaattttccagatc
pET28a-p54 JD	F:aattcgagctcc*GTCGAC***atg**agaaagaaaaaagctg
	R:tggtggtggtggtg*CTCGAG*ttacaaggagtttt
P1	F:ctaccggactc*AGATCT***atg**gatagcgagttttttcag
	R:atcccgggcccgc*GGTACC*gtgctgctgaacagataaa
P2	F:ctaccggactc*AGATCT***atg**cgcaagaaaaaagccgccgcc
	R:atcccgggcccgc*GGTACC*gtcaccggattatcggtaa
P3	F:ctaccggactc*AGATCT***atg**actgatcgtttagttatggc
	R:atcccgggcccgc*GGTACC*gttaaagaattttccagat
P4	F:gcgctaccggactc*AGATCT***atg**actgatcgtttagttatggc
	R:atcccgggcccgc*GGTACC*gtcacggtggtatacggt
P5	F:ctaccggactc*AGATCT***atg**accacccagaataccgccagc
	R:atcccgggcccgc*GGTACC*gttaaagaattttccagat
P6	F:gcgctaccggactc*AGATCT***atg**accacccagaataccg
	R:atcccgggcccgc*GGTACC*gtgtaggtgttgcgctgac
P7	F:ctaccggactc*AGATCT***atg**gatagcgagttttttcag
	R:atcccgggcccgc*GGTACC*gtggtgttgcgctgacg
P8	F:gcgctaccggactc*AGATCT***atg**agccagaccatgagcg
	R:atcccgggcccgc*GGTACC*gtgtaggtgttgcgctga
P9	F:gcgctaccggactc*AGATCT***atg**cagaccatgagcgccat
	R:atcccgggcccgc*GGTACC*gttaaagaattttccagat
P10	F:gcgctaccggactc*AGATCT***atg**accatgagcgccattga
	R:atcccgggcccgc*GGTACC*gttaaagaattttccagat
P11	F:gcgctaccggactc*AGATCT***atg**agcgccattgaaaa
	R:atcccgggcccgc*GGTACC*gttaaagaattttccagat
P12	F:gcgctaccggactc*AGATCT***atg**agcgccattgaaaattt
	R:atcccgggcccgc*GGTACC*gttaaagaattttccagat
P13	F:ctaccggactc*AGATCT***atg**cgcaagaaaaaagccgccgcc
	R:atcccgggcccgcGGTACCgtgctggtaccgggctgc
P14	F:ctaccggactcAGATCT**atg**aaacccgctggtgcaaccacc
	R:atcccgggcccgc*GGTACC*gtcaccggattatcggtaa
P15	F:gcgctaccggactc*AGATCT***atg**cgcaagaaaaaagccg
	R:atcccgggcccgc*GGTACC*gtctgctgatcttggtac
P16	F:ctaccggactc*AGATCT***atg**gatagcgagttttttcag
	R:atcccgggcccgc*GGTACC*gtctgatcttggtacggg
P17	F:ctaccggactc*AGATCT***atg**gatagcgagttttttcag
	R:atcccgggcccgc*GGTACC*gtatcttggtacgggttg
P18	F:ctaccggactc*AGATCT***atg**gatagcgagttttttcag
	R:atcccgggcccgc*GGTACC*gtttggtacgggttgatg
P19	F:ctaccggactc*AGATCT***atg**gatagcgagttttttcag

	R:atcccgggcccgc*GGTACC*gtgtacgggttgatgaac
P20	F:ctaccggactc*AGATCT***atg**gatagcgagttttttcag<br>R:atcccgggcccgc*GGTACC*gtcgggttgatgaactgg
P21	F:ctaccggactc*AGATCT***atg**gatagcgagttttttcag<br>R:atcccgggcccgc*GGTACC*gtgttgatgaactggatg
P22	F:gcgctaccggactc*AGATCT***atg**gccgccatcgaggaag<br>R:atcccgggcccgc*GGTACC*gtctgctgatcttggtac
P23	F:gcgctaccggactc*AGATCT***atg**gccatcgaggaagagga<br>R:atcccgggcccgc*GGTACC*gttaaagaattttccagat
P24	F:aattcgagctcc*GTCGAC***atg**agaaagaaaaaagctg<br>R:atcccgggcccgc*GGTACC*gtttctgccggatgtgccg
P25	F:aattcgagctcc*GTCGAC***atg**agaaagaaaaaagctg<br>R:atcccgggcccgc*GGTACC*gttgccggatgtgccggtg
P26	F:gcgctaccggactc*AGATCT***atg**actgatcgtttagttat<br>R:atcccgggcccgc*GGTACC*gtttctgccggatgtgcc
P27	F:gcgctaccggactc*AGATCT***atg**gcaaccggtggtcc<br>R:atcccgggcccgc*GGTACC*gtgctgctgaacagataaa
P28	F:gcgctaccggactc*AGATCT***atg**gcaaccggtggtcccgc<br>R:atcccgggcccgc*GGTACC*gtgctgctgaacagataaa

Note: All the amplified PCR fragments were ligated into vectors by Seamless Cloning/In-Fusion Cloning. F, forward; R, reverse. The restriction sites are in italic, and the start codons are marked in bold.
