# Supplementary figures and images for: The Novel Antigenic Epitopes of African Swine Fever Virus Inner Membrane p54 Protein Revealed by Monoclonal Antibodies
Source: Animals (Basel). 2025 Apr 30;15(9):1296. doi: 10.3390/ani15091296 (PMC12070866; doi:10.3390/ani15091296)

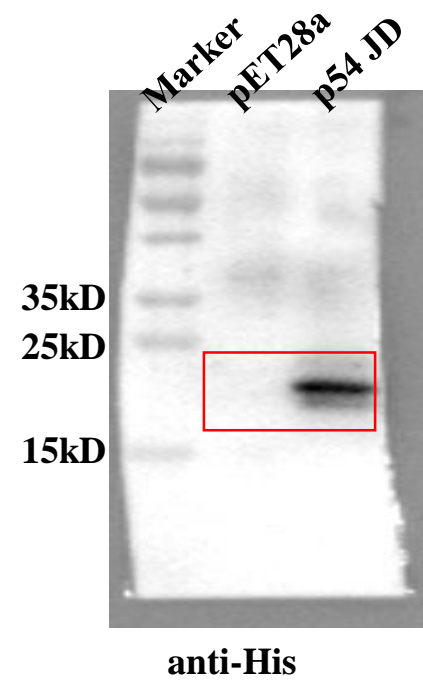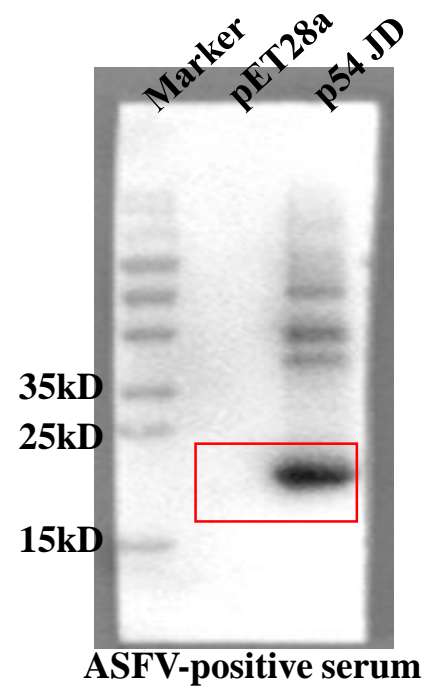

Fig 1

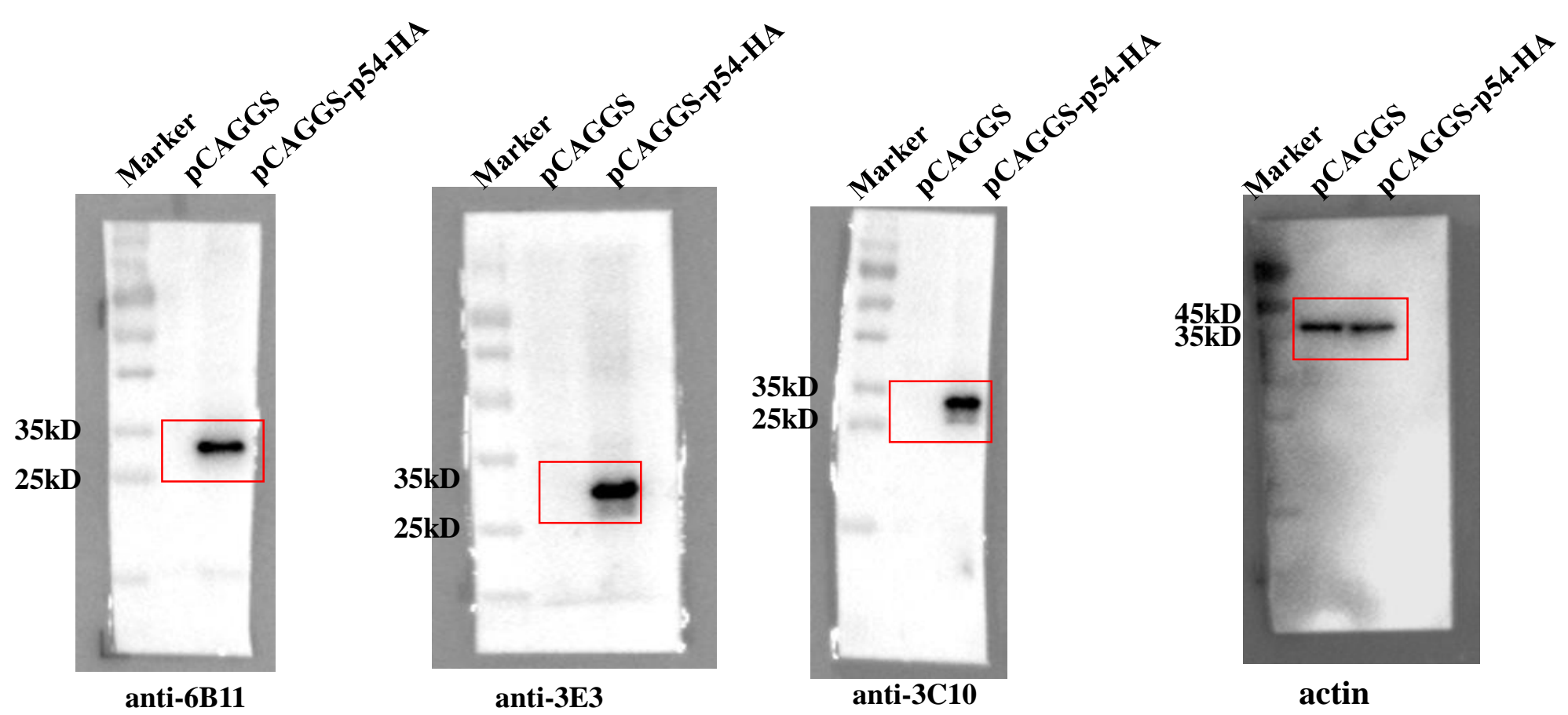

Fig 3A

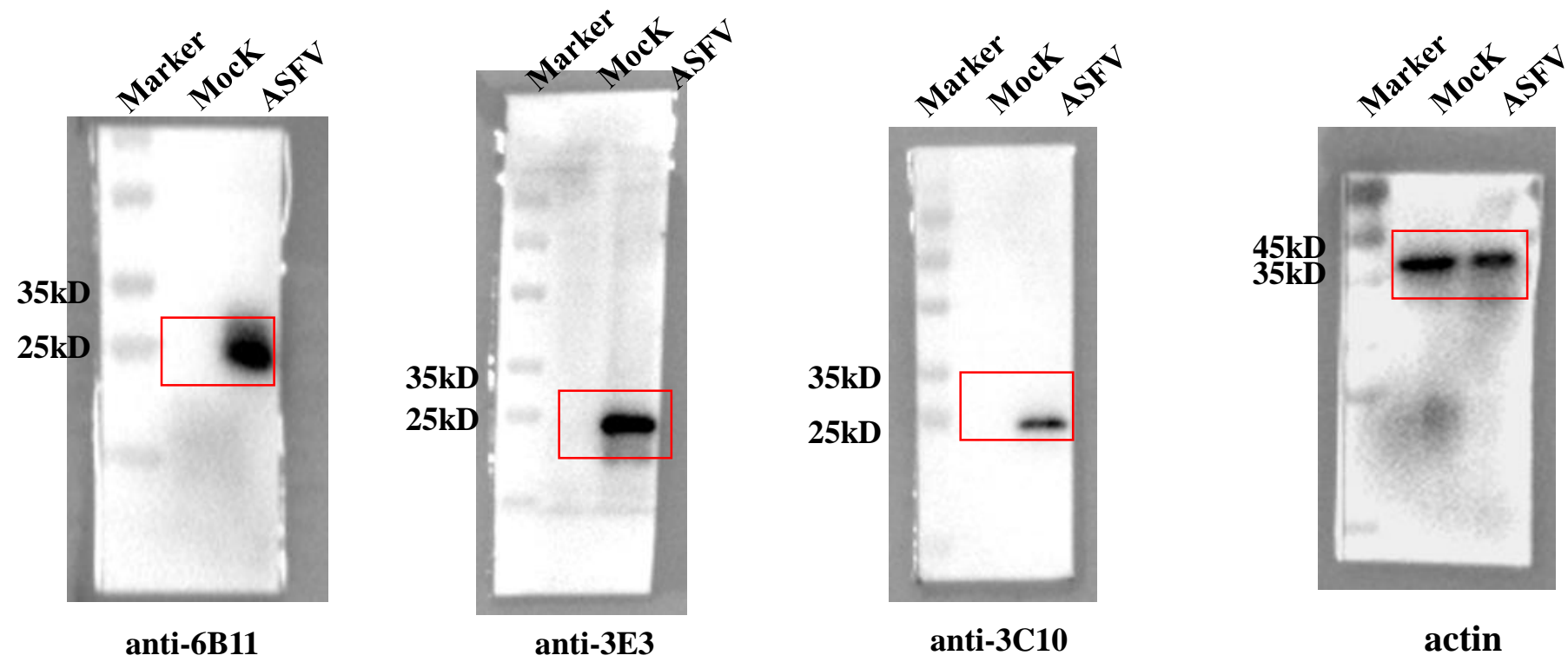

Fig 3B

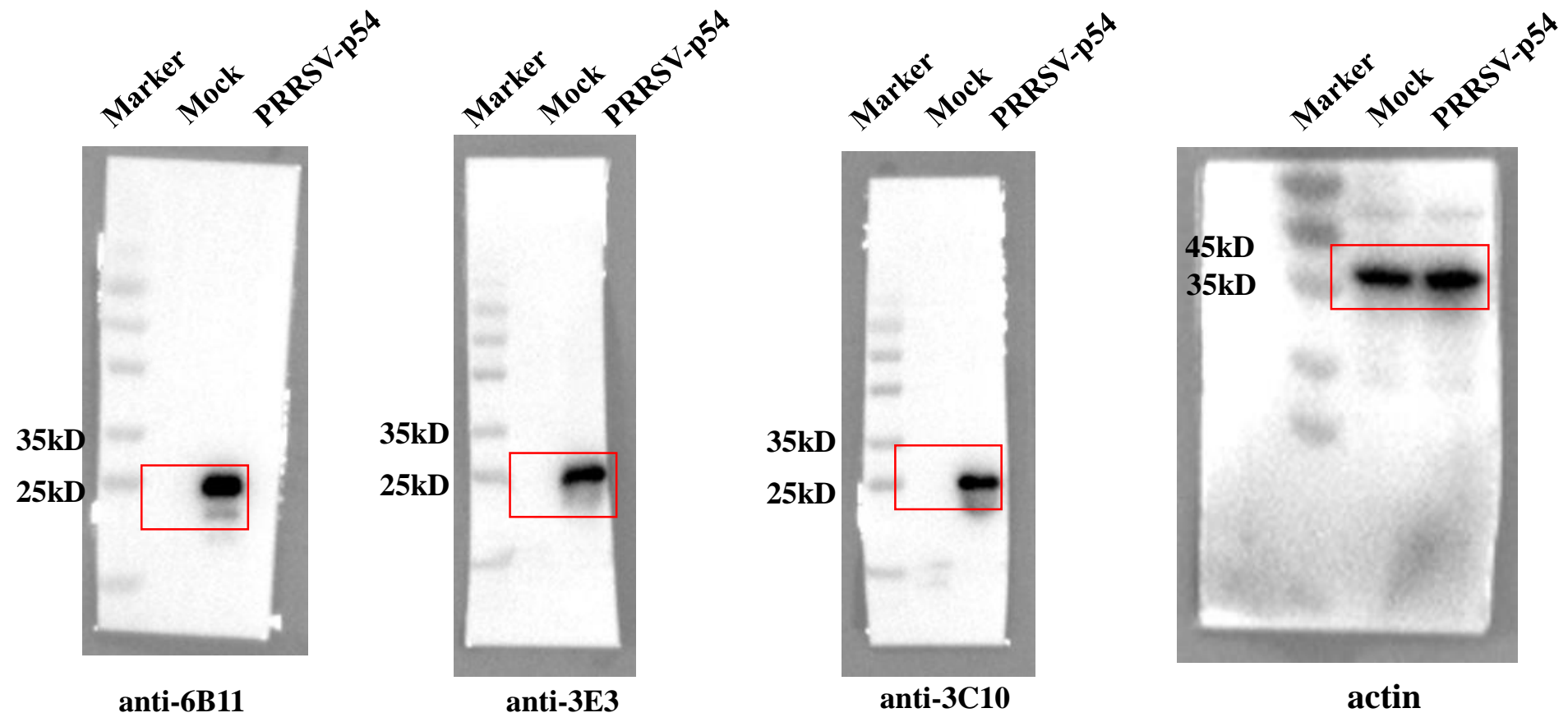

Fig 3C

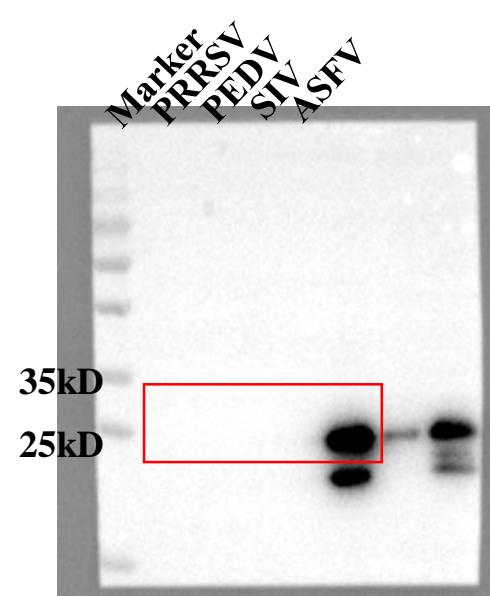

anti-6B11

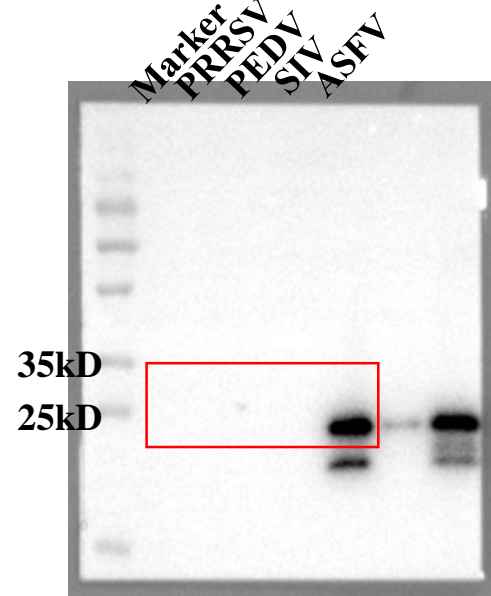

anti-3E3

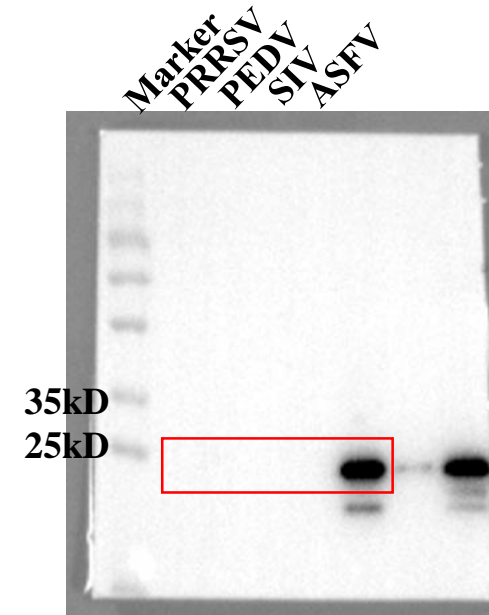

anti-3C10

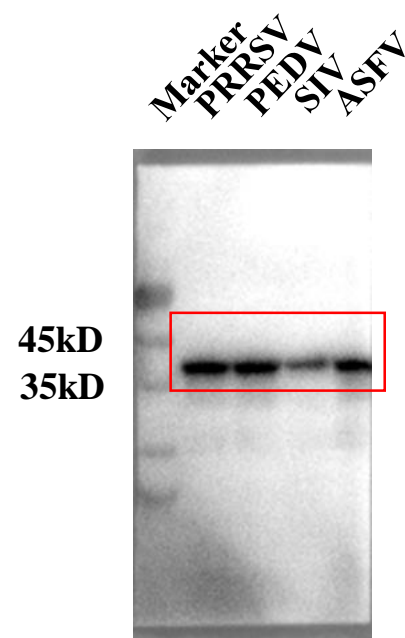

actin

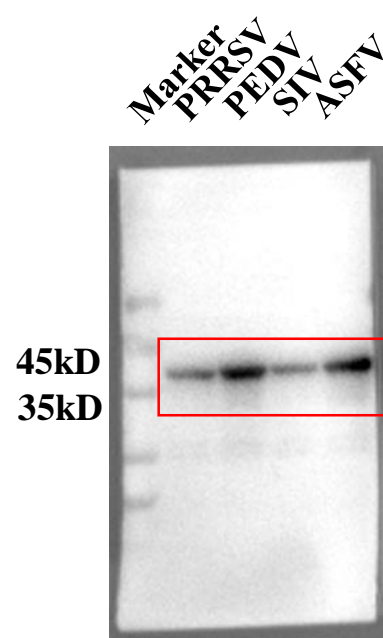

actin

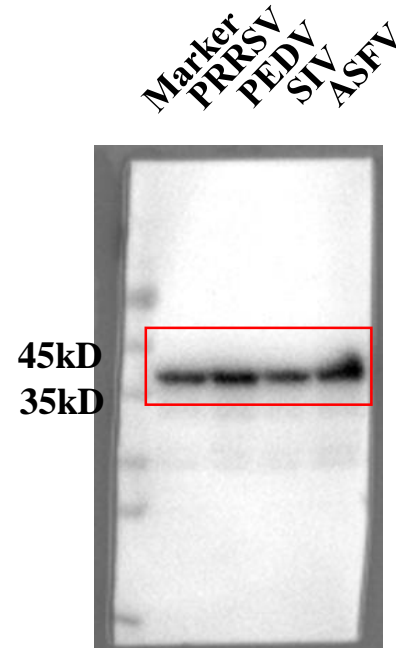

actin

Fig 3D-F

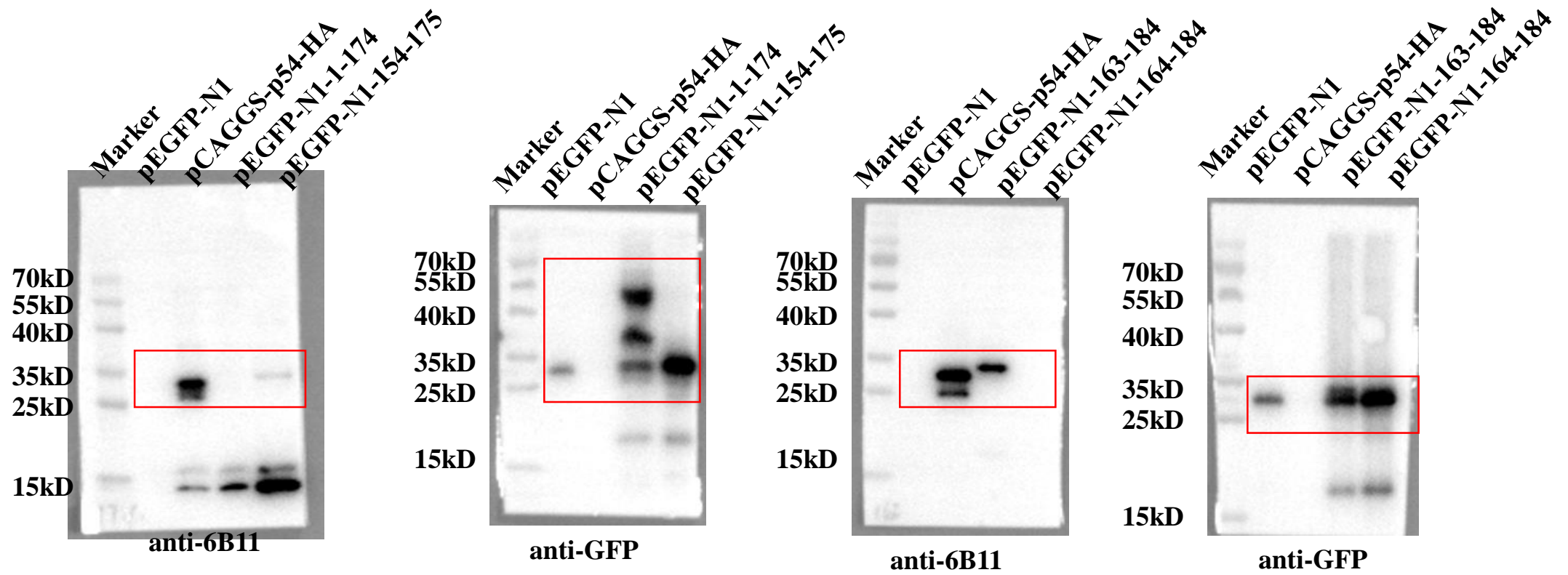

Fig 5 up

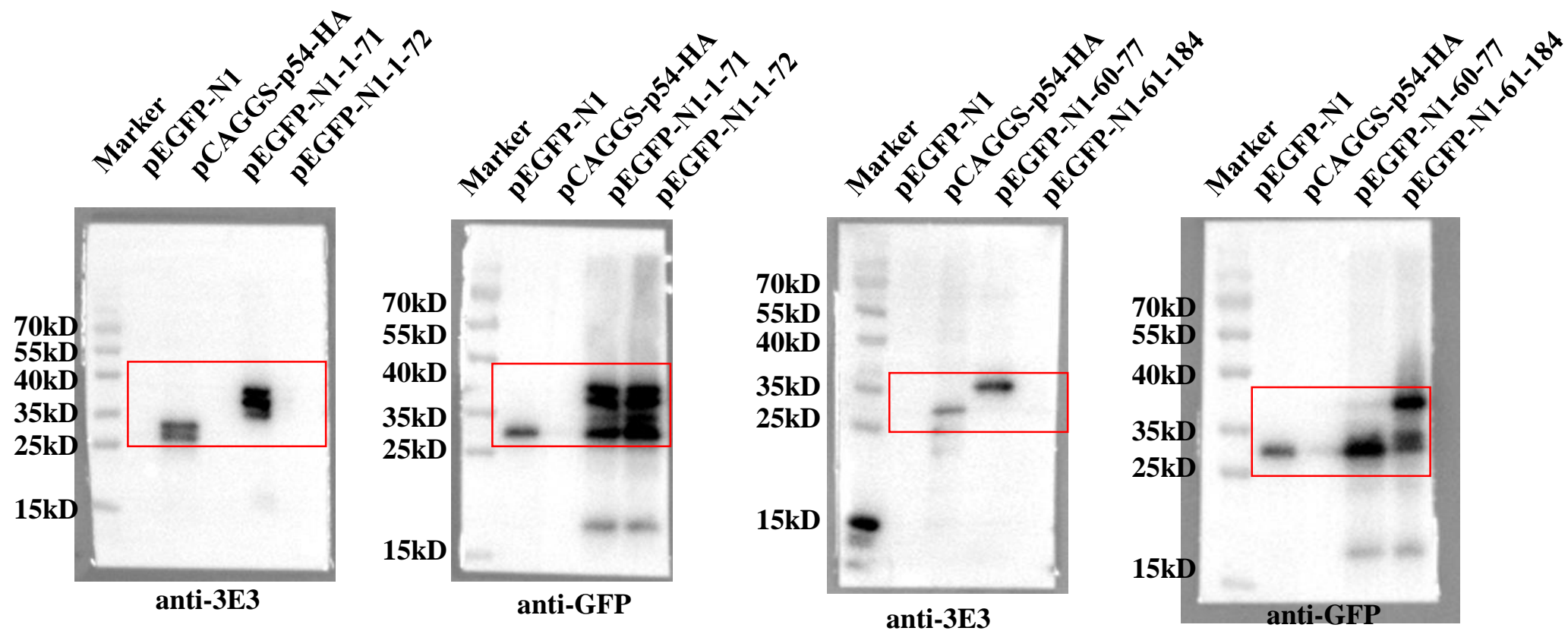

Fig 5 middle

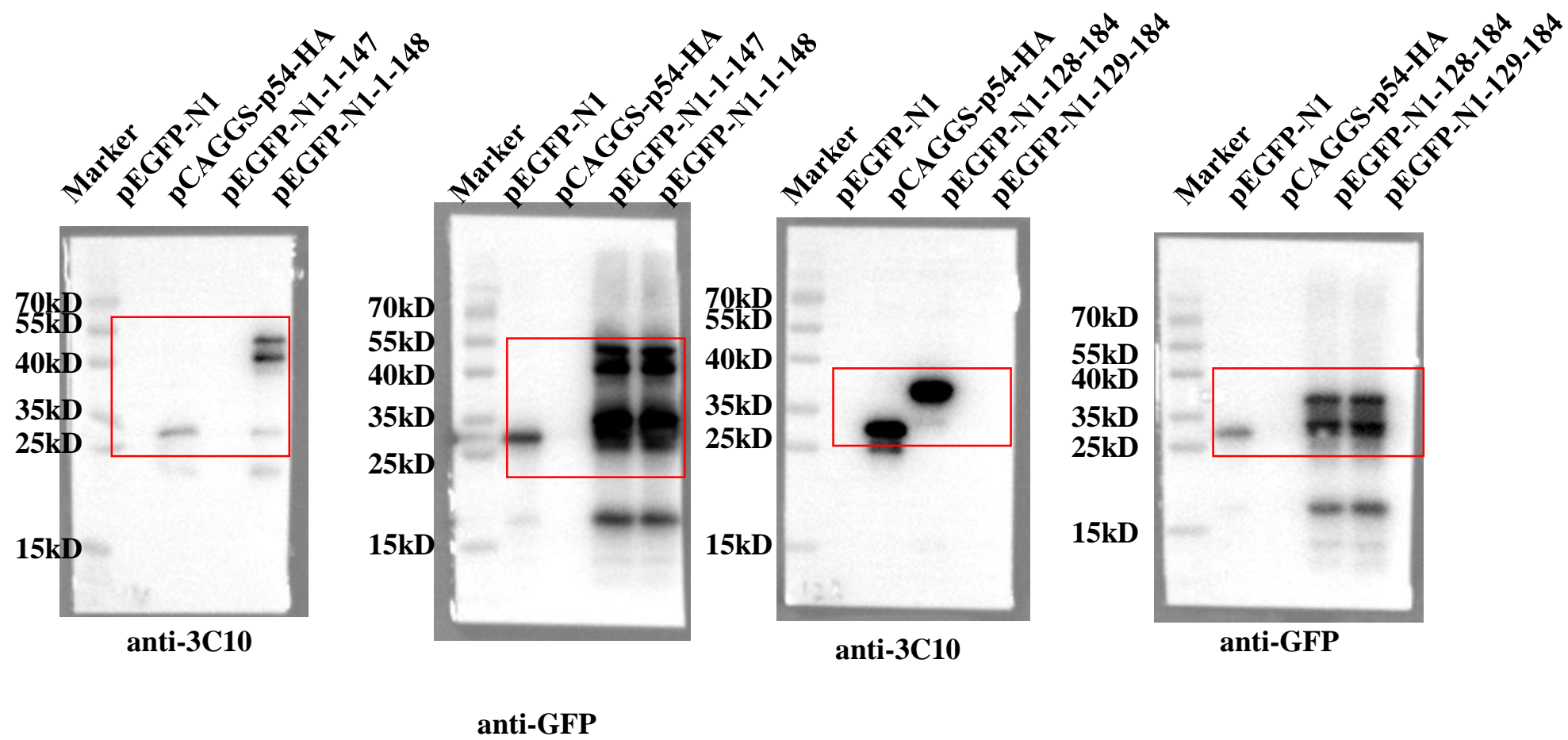

Fig 5 low

Supplement: Supplementary file 1 [file animals-15-01296-s001.zip › WB raw data.pdf]
